# Vestibular schwannomas: Accuracy of tumor volume estimated by ice cream cone formula using thin-sliced MR images

**DOI:** 10.1371/journal.pone.0192411

**Published:** 2018-02-13

**Authors:** Hsing-Hao Ho, Ya-Hui Li, Jih-Chin Lee, Chih-Wei Wang, Yi-Lin Yu, Dueng-Yuan Hueng, Hsin-I Ma, Hsian-He Hsu, Chun-Jung Juan

**Affiliations:** 1 Department of Radiology, Tri-Service General Hospital, Taipei, Taiwan; 2 Department of Radiology, National Defense Medical Center, Taipei, Taiwan; 3 Department of Otolaryngology-Head and Neck Surgery, Tri-Service General Hospital, Taipei, Taiwan; 4 Department of Otolaryngology-Head and Neck Surgery, National Defense Medical Center, Taipei, Taiwan; 5 Department of Neurological Surgery, Tri-Service General Hospital, Taipei, Taiwan; 6 Department of Neurological Surgery, National Defense Medical Center, Taipei, Taiwan; 7 Department of Biochemistry, National Defense Medical Center, Taipei, Taiwan; University of Iowa, UNITED STATES

## Abstract

**Purpose:**

We estimated the volume of vestibular schwannomas by an ice cream cone formula using thin-sliced magnetic resonance images (MRI) and compared the estimation accuracy among different estimating formulas and between different models.

**Methods:**

The study was approved by a local institutional review board. A total of 100 patients with vestibular schwannomas examined by MRI between January 2011 and November 2015 were enrolled retrospectively. Informed consent was waived. Volumes of vestibular schwannomas were estimated by cuboidal, ellipsoidal, and spherical formulas based on a one-component model, and cuboidal, ellipsoidal, Linskey’s, and ice cream cone formulas based on a two-component model. The estimated volumes were compared to the volumes measured by planimetry. Intraobserver reproducibility and interobserver agreement was tested. Estimation error, including absolute percentage error (APE) and percentage error (PE), was calculated. Statistical analysis included intraclass correlation coefficient (ICC), linear regression analysis, one-way analysis of variance, and paired t-tests with *P* < 0.05 considered statistically significant.

**Results:**

Overall tumor size was 4.80 ± 6.8 mL (mean ±standard deviation). All ICCs were no less than 0.992, suggestive of high intraobserver reproducibility and high interobserver agreement. Cuboidal formulas significantly overestimated the tumor volume by a factor of 1.9 to 2.4 (*P* ≤ 0.001). The one-component ellipsoidal and spherical formulas overestimated the tumor volume with an APE of 20.3% and 29.2%, respectively. The two-component ice cream cone method, and ellipsoidal and Linskey’s formulas significantly reduced the APE to 11.0%, 10.1%, and 12.5%, respectively (all *P* < 0.001).

**Conclusion:**

The ice cream cone method and other two-component formulas including the ellipsoidal and Linskey’s formulas allow for estimation of vestibular schwannoma volume more accurately than all one-component formulas.

## Introduction

Vestibular schwannomas are benign tumors that arise most commonly from the nerve sheath of the vestibular portion of cranial nerve VIII [1]. Size of the vestibular schwannomas is a factor influencing the choice of treatment [2–5]. Small vestibular schwannomas can be either managed conservatively [6] or treated by radiosurgery [3, 7], while large vestibular schwannomas often require surgical intervention. According to the International RadioSurgery Association (IRSA) guidelines, in general, radiosurgery is effective for vestibular schwannomas less than 3 cm in diameter, while microsurgery is the first choice for vestibular schwannomas larger than 3 cm in diameter [8]. Tumor size has been used as an important prognostic factor for preserving cochlear and facial nerve function [5, 9, 10]. Change of tumor size has been used as an indicator of treatment response [11]. Moreover, it must be followed in patients receiving either conservative or aggressive treatments [10, 12].

In vestibular schwannomas, tumor volume can be measured based on slice-by-slice planimetry [13, 14]. Although planimetry has been regarded as the standard method in measuring vestibular schwannoma volume, it is rather time-consuming [15] and not convenient in daily practice. Alternatively, the tumor size can be estimated rapidly based on cuboidal [16, 17], spherical [18], or ellipsoidal [13–15, 17, 19–23] formulas based on either one- [16, 19, 23] or two- [18, 24–28] component model. Despite the increasing use of volume-estimating formulas, estimation accuracy among these formulas has, to our knowledge, never been verified in a single study to date. We hypothesized that the estimation accuracy will be different among formulas based on different assumption of shapes and be different between one- and two-component models. We estimated the volume of vestibular schwannomas by an ice cream cone formula using thin-sliced MRIs and compared the estimation accuracy among different estimating formulas and between different models.

## Materials and methods

### Patient population

This retrospective study was approved by the institutional review board of the Tri-Service General Hospital. Written informed consent was waived because of the retrospective nature. Between January 2011 and November 2015, a total of 137 patients diagnosed with vestibular schwannoma underwent brain magnetic resonance imaging (MRI) at our hospital. Patients with bilateral lesions (*n* = 2), solely intracanalicular lesions (*n* = 20), or ill-defined margins (*n* = 3), or without thin slice sequences (*n* = 12) were excluded (S1 Fig). Finally, a total of 100 patients were enrolled into this study (41 men and 59 women; age range, 22–92 years; mean age ± standard deviation, 54.7 ± 14.8 years). Demographic characteristics of the 100 patients are shown in Table 1.

**Table 1:** Demographic characteristics of 100 patients with vestibular schwannoma.

Age (years)	Mean	54.8
	Maximal	92
	Minimal	22
Sex	Male	41
	Female	59
Lesion side	Right	49
	Left	51
Groups of tumor volume	Small (<1.0 mL)	34
	Medium (1.0~4.0 mL)	33
	Large (>4.0 mL)	33

### Image acquisition

MRI protocols for the study patients were shown in S1 Table. Tumor volume was measured in the axial plane on thin-slice contrast-enhanced T1-weighted images (T1WI) in 83 patients and on either fast imaging employing steady-state acquisition-cycled phases (FIESTA-C) or balanced fast field echo sequences (bFFE) in the others. Slice thickness ranged from 0.8 to 2 mm, including 16 2D images with a slice thickness of 0.9 to 2 mm with zero gap, and 84 3D images with a slice thickness of 0.8 to 1.4 mm zero interpolated (ZIP) by a factor of 2 to approximately 0.4 to 0.7 mm.

### Tumor volume calculation

All measurements were performed using a picture archiving and communication system (PACS; EBM Technology, Taipei, Taiwan). Tumor volume calculated by planimetry was regard as standard in this study. The tumor was outlined manually slice-by-slice using polygonal region-of-interest (ROI). The area of each ROI was calculated automatically by the PACS viewer (EBM viewer; EBM Technology). The tumor volume then was calculated by multiplying the summation of the areas by the slice thickness.

**Fig 1:**
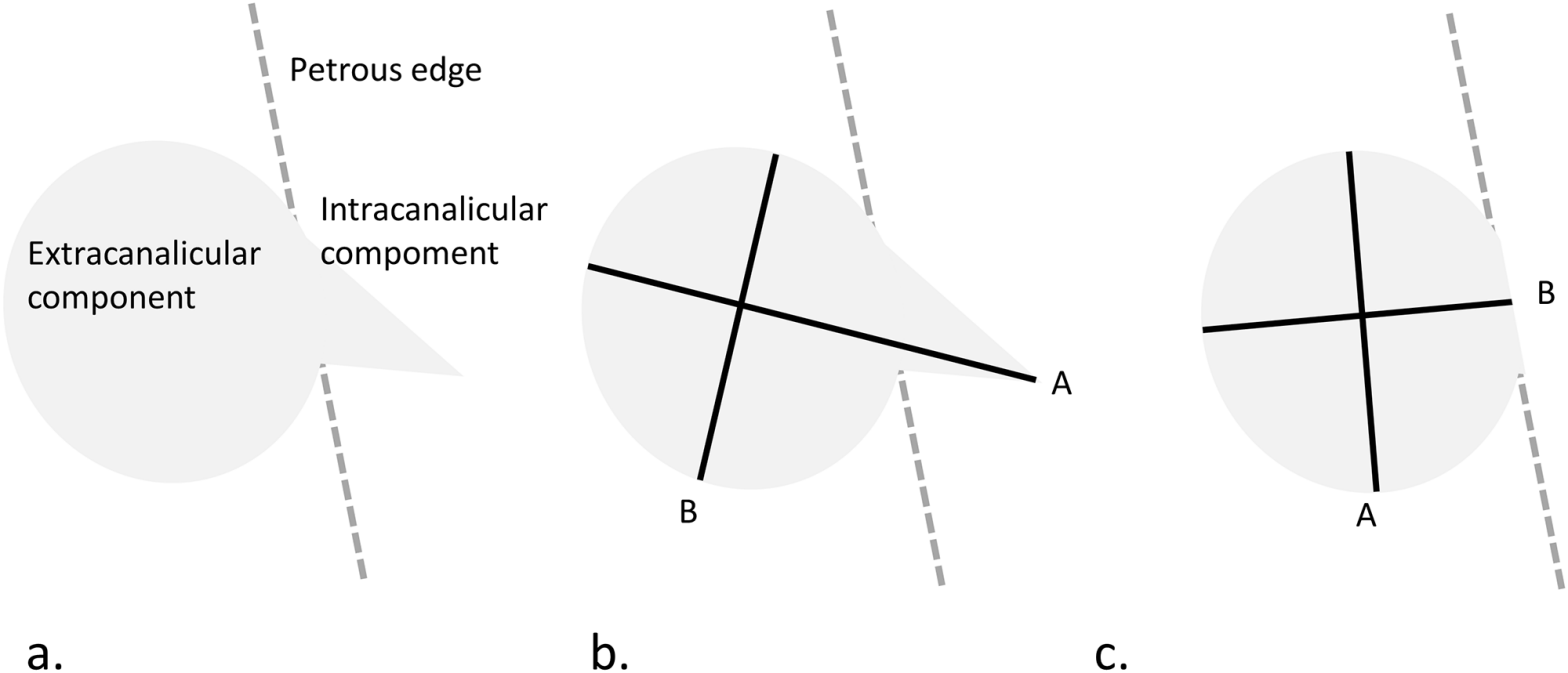
Cartoon illustration of one-component formulas on the axial plane. (a) A vestibular schwannoma is divided conceptually into intra- and extracanalicular components. (b) The intra- and extracanalicular components are included in the volume calculation. (c) Only the extracanalicular component is included in the volume calculation. In (b) and (c), parameter A is the largest diameter of the tumor on the axial plane, parameter B is the largest diameter perpendicular to parameter A on the axial plane, and parameter C (not shown in this Fig) is the height calculated by the product of slice thickness and the number of slices containing the tumor.

**Fig 2:**
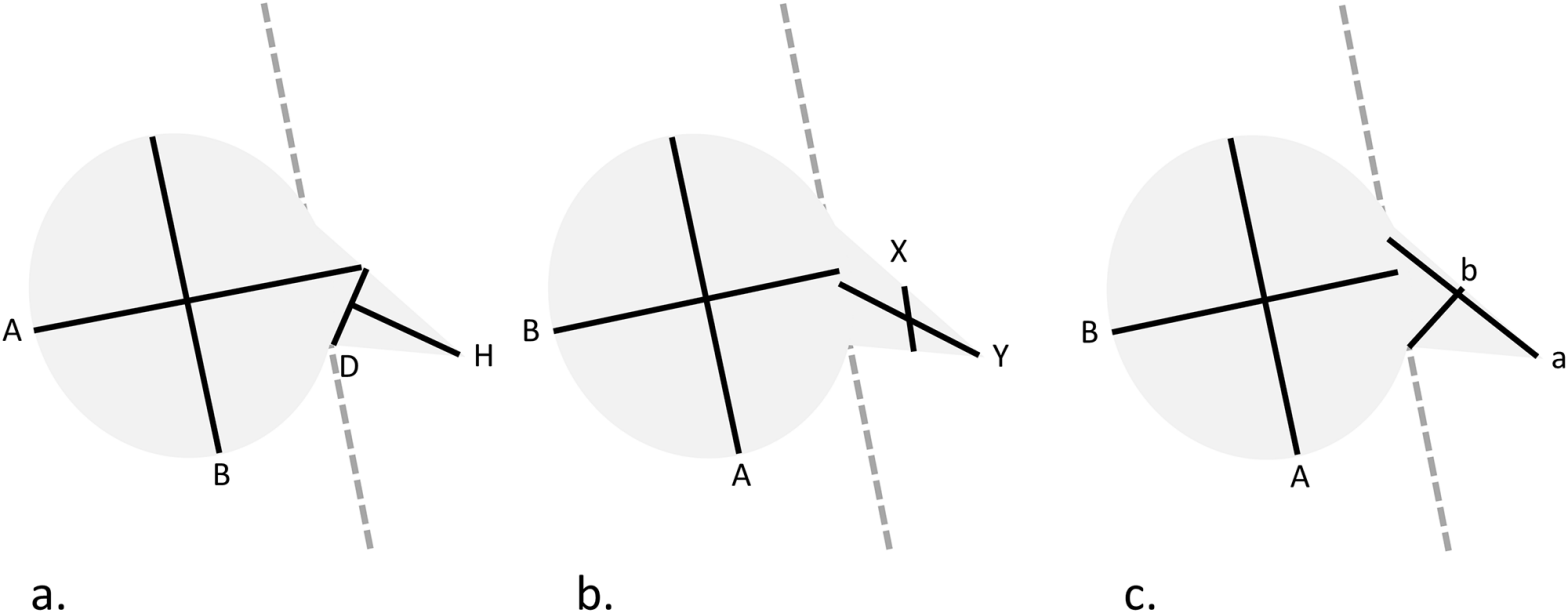
Cartoon illustration of two-component formulas on the axial plane. (a) In the ice cream cone formula, the tumor is separated into cone and ball components. An axial line is drawn along the axis of the internal acoustic canal (IAC), and then a base line is drawn from the posterior margin of the internal orifice of the IAC and perpendicular to the axial line. The height of the cone (parameter H) is derived from the axial line, and the diameter of the base of the cone (parameter D) is derived from the base line. (b) In Linskey’s method, parameter X represents the cross-sectional diameter of the midpoint of the bony edges of the IAC, and parameter Y is the length of the IAC. (c) In the two-component ellipsoidal or cuboidal method, parameter “a” is the longest dimension of the intracanalicular component, parameter “b” is perpendicular to “a,” and parameter “c” (not shown in this figure) is the height of the intracanalicular component.

The tumor volume was estimated based on one- and two-component models, respectively. Cartoon illustrations of a vestibular schwannoma with measurements for the tumor volume estimation in the one- and two-component models were depicted in Figs 1 and 2, respectively. Conceptually, the vestibular schwannoma was separated into extracanalicular and intracanalicular components. In the one-component model, the tumor volume was estimated for the whole tumor and solely the extracanalicular component using cuboidal, ellipsoidal, and spherical formulas, respectively. In the two-component model, the volumes of extra- and intracanalicular components were estimated independently using ice cream cone, cuboid, ellipsoid, and Linskey’s formula, respectively. All formulas used for tumor volume estimation were demonstrated in Table 2.

**Table 2:** Formulas to estimate tumor volume based on the one- and two-component models.

Model	Assumption	Formula
One-component	Cuboidal	V = ABC
	Ellipsoidal	$\text{V}=\frac{4}{3}\pi \left(\frac{A}{2}\right)\left(\frac{B}{2}\right)\left(\frac{C}{2}\right)=\left(\frac{\pi }{3}\right)\left(\frac{ABC}{2}\right)≒\frac{ABC}{2}$
	Spherical	${\text{D}}_{\text{a}}=\frac{A+B+C}{3}$
		$\text{V}=\frac{4}{3}\pi {\left(\frac{D_{a}}{2}\right)}^{3}=\left(\frac{\pi }{3}\right)\left(\frac{D_{a}^{3}}{2}\right)≒\frac{D_{a}^{3}}{2}$
Two-component	Ice cream cone	${\text{V}}_{\text{ic}}=\frac{1}{3}\pi r^{2}H=\frac{1}{3}\pi {\left(\frac{D}{2}\right)}^{2}H=\left(\frac{\pi }{3}\right)\left(\frac{D^{2}H}{4}\right)≒\frac{D^{2}H}{4}$
		${\text{V}}_{\text{ec}}=\frac{\text{ABC}}{2}$
	Spherical + ellipsoidal (Linskey’s)	${\text{V}}_{\text{ic}}=\frac{4}{3}\pi \left(\frac{X}{2}\right)\left(\frac{X}{2}\right)\left(\frac{Y}{2}\right)=\frac{\pi X^{2}Y}{6}$
		${\text{D}}_{\text{a}}=\frac{A+B+C}{3}$
		${\text{V}}_{\text{ec}}=\frac{4}{3}\pi {\left(\frac{D_{a}}{2}\right)}^{3}=\frac{\pi D_{a}^{3}}{6}$
	Cuboidal + Cuboidal	V_ic_ = abc V_ec_ = ABC
	Ellipsoidal + Ellipsoidal	${\text{V}}_{\text{ic}}=\frac{\text{abc}}{2}$
		${\text{V}}_{\text{ec}}=\frac{\text{ABC}}{2}$

Note: A, maximal diameter of the tumor on axial image; abc, length, width, and height of the intracanalicular component of the tumor; B, the maximal diameter orthogonal to A on the axial image; C, height of the tumor calculated by the product of slice thickness and the number of slices containing the tumor; D_a_, averaged diameter; D, diameter of base of the “cone portion”; H, height of the “cone portion”; V, tumor volume; V_ic_, volume of intracanalicular component of tumor; V_ec_, volume of extracanalicular component of tumor; X, cross-sectional diameter of midpoint of the intracanalicular canal; Y, length of the intracanalicular canal.

The tumor volume estimated by each of the aforementioned formulas was generated automatically by Microsoft Excel 2010 after manually keying in parameters including slice thickness, slice number, diameters, and the area of each slice containing the tumor.

### Intraobserver reproducibility and interobserver agreement

Tumor volume was estimated independently by two observers under the supervision of C.J.J. (more than 10 years of experience in neuroradiology). Observer 1 (H.H.H., four years of radiology residency) performed all measurements twice at an interval of three months to examine the intraobserver reproducibility. Observer 2 (Y.H.L., three years of radiology residency) only estimated the tumor volume by the ABC/2 formula and the ice cream cone method once in 30 randomly selected cases to examine the interobserver agreement.

### Analysis of estimation error

Estimation errors were expressed by percentage error (PE), which was defined as [(estimated volume—standard volume) / standard volume] ×100%, and absolute percentage error (APE), which was defined as absolute value of PE. Mean PE and mean APE were used to represent the bias and prediction accuracy of measurements, respectively.

### Statistical analyses

Statistical analyses were performed using SPSS 16.0 (SPSS, Inc., Chicago, IL, USA). Linear regression analysis was used to evaluate the relationship between all estimating formulas and planimetry. Intraclass correlation coefficients (ICC) and Bland-Altman plots were applied to evaluate the intraobserver reproducibility and interobserver agreement. One-way analysis of variance plus post hoc analysis with Tukey correction and paired *t*-test were used for group comparisons. *P* < 0.05 was defined as statistically significant.

## Results

### Standard and estimated tumor volumes

The tumor volumes calculated by planimetry and estimated by different formulas based on one- and two-component models were shown in Table 3. Cuboidal formulas significantly overestimated the tumor volume relative to planimetry (4.8 mL) no matter based on the one-component (11.75 mL using the whole tumor for estimation, *P* < 0.001; 9.32 mL using the extracanalicular component to represent the tumor, *P* = 0.007) or two-component (10.26 mL, *P* = 0.001) model. Estimates obtained from the other formulas did not differ from planimetry regarding the tumor volume. Linear regression analysis showed a significantly positive correlation between the estimated tumor volume using any estimating formula and the standard tumor volume (all *R*^2^ > 0.980; *P* < 0.001).

**Table 3:** Tumor volumes measured by planimetry and estimated by one- and two-component formulas.

Model	Formula	Volume	Mean volume	SD	P value	Mean APE	SD	Mean PE	SD
Planimetry		V_t_	4.8	6.78					
One-component	C	V_t_	11.75	16.6	<0.001	140.5	31.0	140.5	31.0
		V_ec_	9.32	15.11	0.007	65.2	36.2	57.1	48.1
	E	V_t_	5.88	8.3	0.893	21.8	13.2	20.3	15.5
		V_ec_	4.66	7.55	1.000	24.7	20.7	-21.5	24.0
	S	V_t_	6.05	8.45	0.845	29.2	17.3	28.6	18.2
		V_ec_	4.84	7.8	1.000	22.0	19.9	-17.3	24.2
Two-component	Ice cream cone	V_t_	5.25	7.85	0.998	11.0	10.5	1.6	15.1
	Linskey’s (S+E)	V_t_	5.43	8.26	0.991	12.4	10.6	7.1	14.8
	C+C	V_t_	10.26	15.35	0.001	111.4	23.9	111.4	23.9
	E+E	V_t_	5.13	7.68	0.999	10.0	8.6	5.7	11.9

APE, absolute percentage error; C, cuboidal; E, ellipsoidal; PE, percentage error; S, spherical; SD, standard deviation; V_t_, total tumor volume; V_ec_, volume of extracanalicular component of tumor.

### Intraobserver reproducibility and interobserver agreement

**Fig 3:**
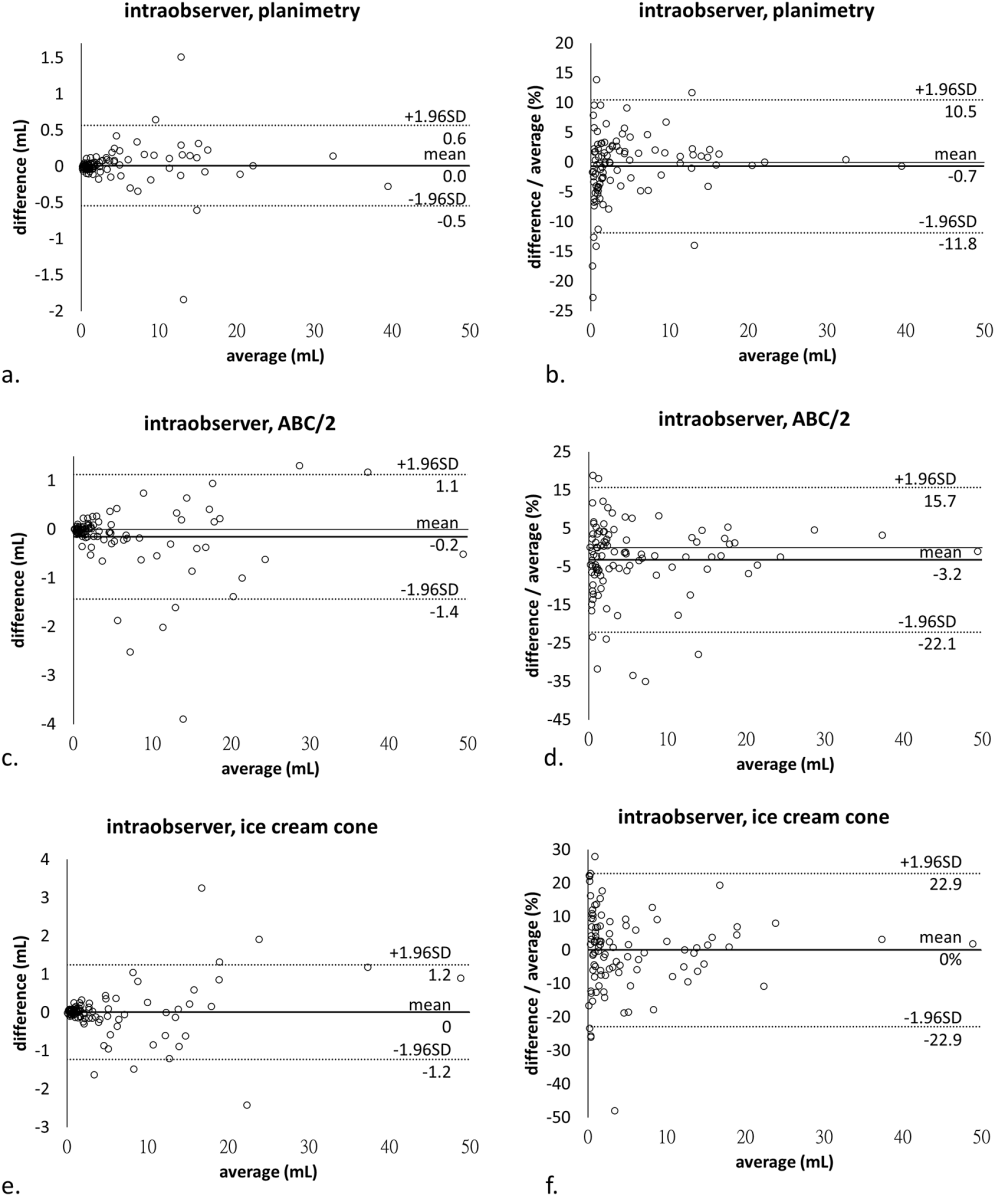
Bland-Altman plots demonstrate intraobserver reproducibility of (a, b) planimetry, and the (c, d) ellipsoidal and (e, f) ice cream cone formulas, with difference and difference-to-average ratio as the Y axis, respectively. Note: SD, standard deviation.

**Fig 4:**
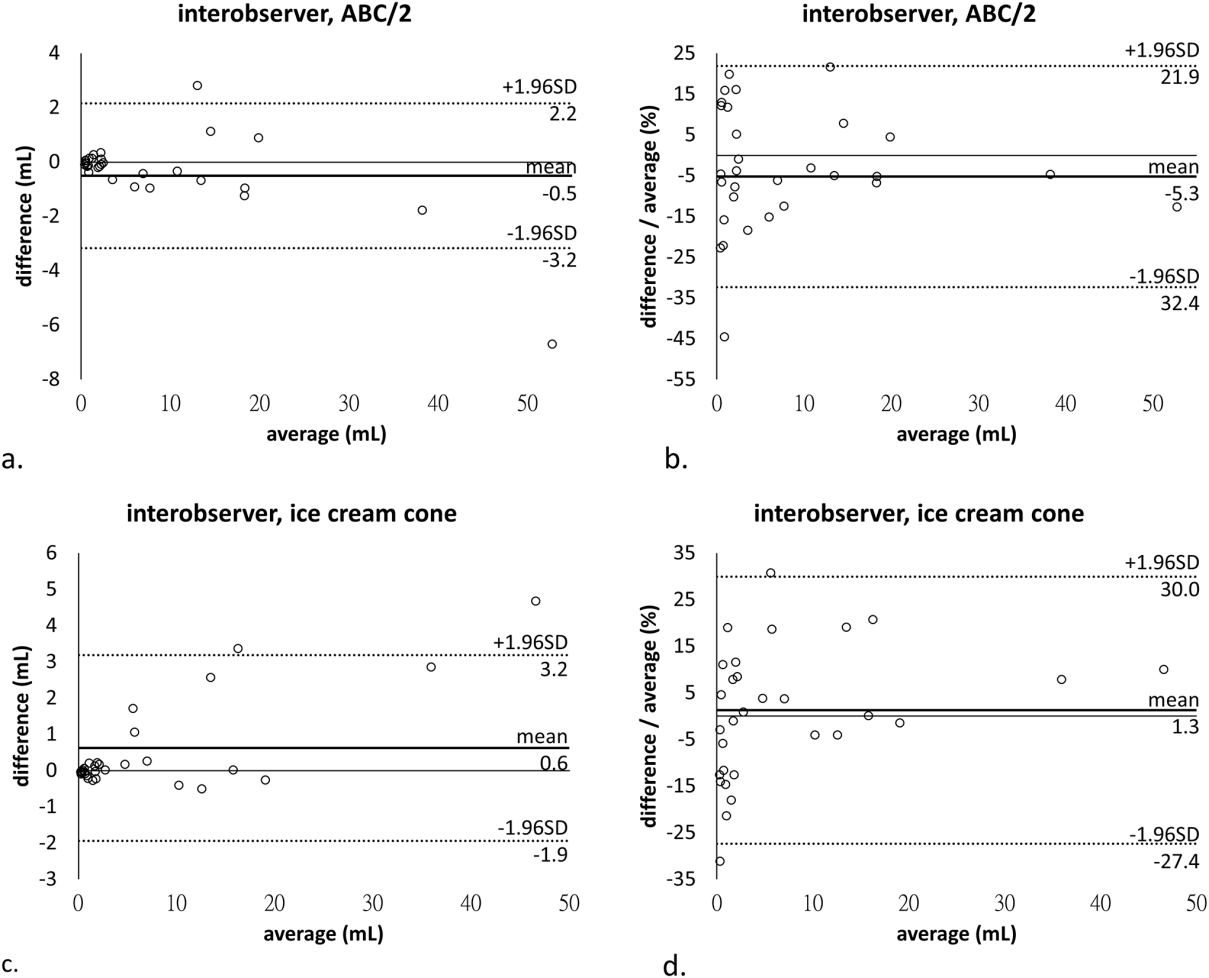
Bland-Altman plots demonstrate interobserver agreement of the (a, b) ellipsoidal and (c, d) ice cream cone formulas, with difference and difference-to-average ratio as the Y axis, respectively. Note: SD, standard deviation.

On Bland-Altman plots (Figs 3 and 4), all data fell within the acceptable ranges and most of them were close to zero. In relative quantification, 95% limits of agreement shown as average difference ± 1.96 standard deviation of the difference were -11.8% to 10.5% (-0.7 ± 11.2%) for planimetry, -22.1% to 15.7% (-3.2 ± 18.9%) for the ABC/2 formula, and -22.9% to 22.9% (0.0 ± 22.9%) for the ice cream cone method in intraobserver analysis, as well as -32.4% to 21.9% (-5.3 ± 27.1%) for the ABC/2 formula and -27.4% to 30.0% (1.3 ± 28.7%) for the ice cream cone method in interobserver analysis. There was a trend of higher difference in large-sized tumors and higher difference-to-average ratio in small-sized tumors.

ICCs of the tumor volume between the first and second measurements of observer 1 were 0.999, 0.997, and 0.997 for planimetry, and the ellipsoidal and ice cream cone formulas, respectively. The ICCs of the tumor volume between the measurements of observers 1 and 2 were 0.992 for both ellipsoidal and ice cream cone formulas. Bland-Altman plots and ICCs suggested high intraobserver reproducibility and high interobserver agreement.

### APE and PE

**Fig 5:**
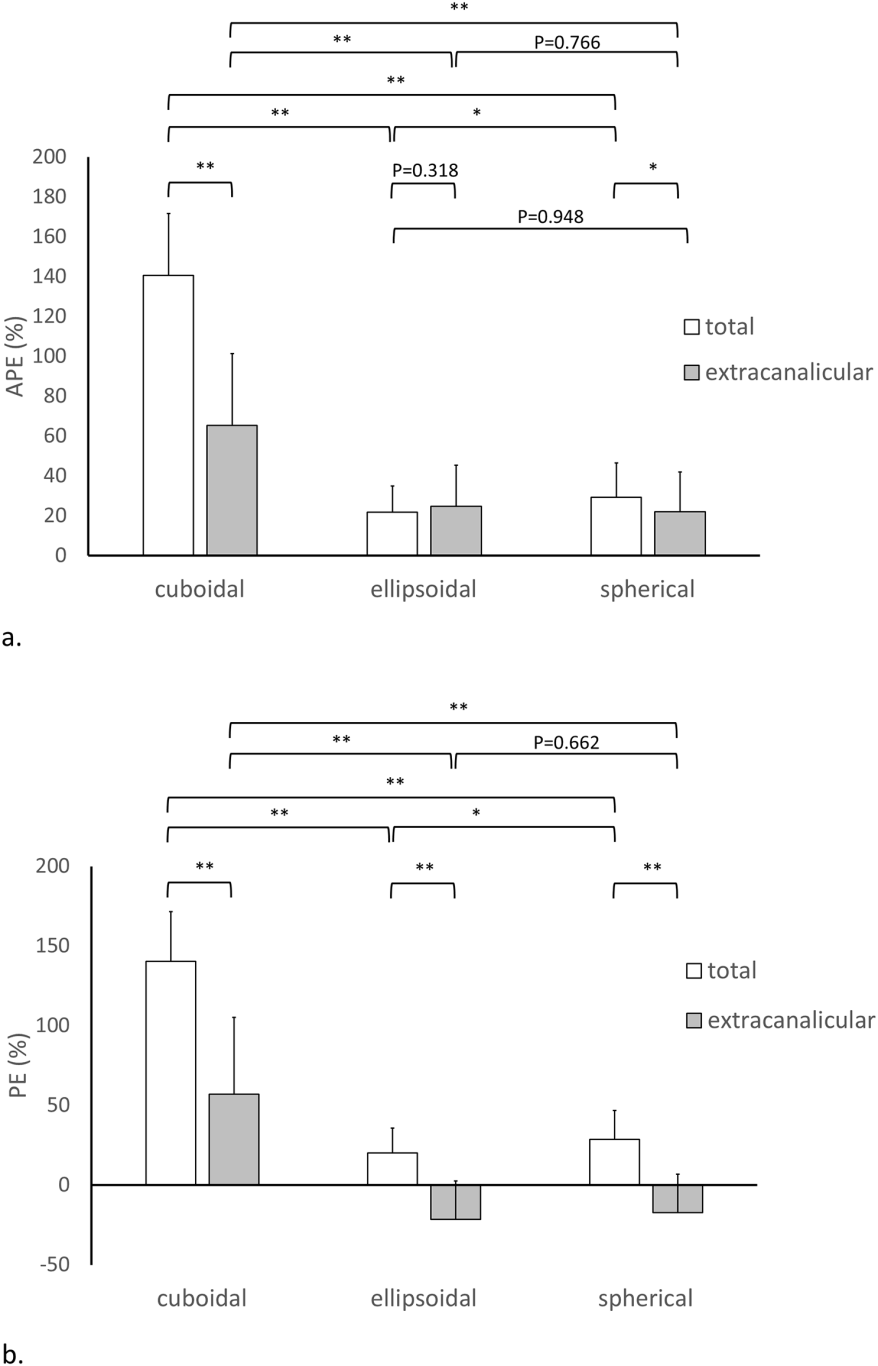
Comparisons of estimation errors among cuboidal, ellipsoidal, and spherical formulas based on one-component model regarding (a) APE and (b) PE. Note: *P < 0.05; **P < 0.001.

**Fig 6:**
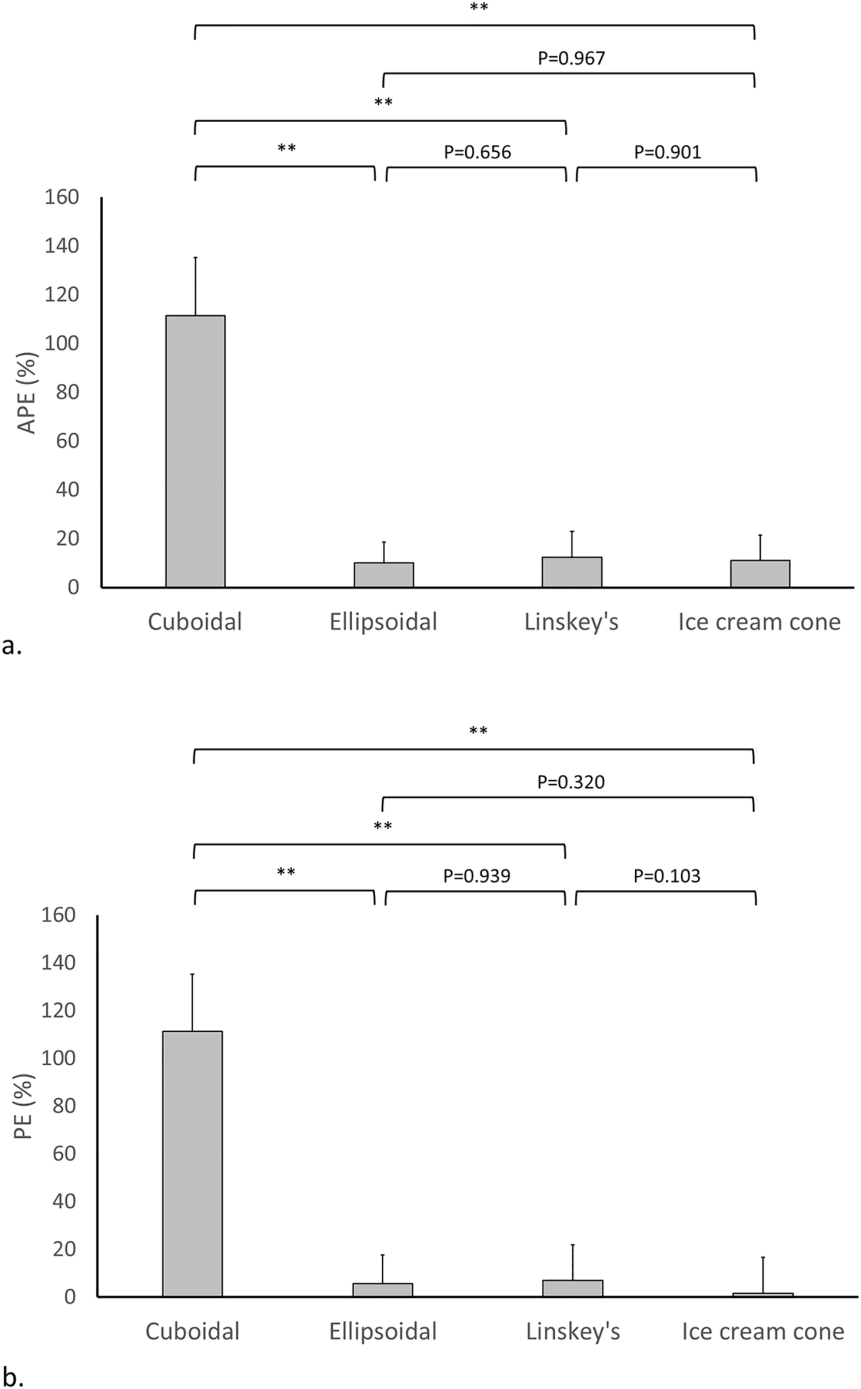
Comparisons of estimation errors among cuboidal, ellipsoidal, Linskey’s and ice cream cone formulas based on two-component model regarding (a) APE and (b) PE. Note: *P < 0.05; **P < 0.001.

Estimation errors of all formulas based on one- and two-component models were shown in Figs 5 and 6, respectively. In the one-component model, all formulas demonstrated a positive mean PE using the whole tumor for calculation, suggesting overestimation of the tumor volume. When the extracanalicular component was used to represent the tumor for calculation, the cuboidal formula still had a positive mean PE, while the ellipsoidal and spherical formulas showed a negative mean PE. This finding suggested that the cuboidal formula overestimated the tumor volume, while the ellipsoidal and spherical formulas underestimated the tumor volume. The ellipsoidal formula showed significantly lower APE and PE than the spherical formula regardless of whether the whole tumor was used for calculation or not (*P* < 0.05). The ellipsoidal formula did not differ from the spherical formula in either APE or PE (*P* = 0.66–0.77) using the extracanalicular component for calculation. On the other hand, the cuboidal formula showed significantly higher APE than the ellipsoidal and spherical formulas regardless of whether the whole tumor or the extracanalicular component was used for calculation (*P* < 0.001).

PE of the cuboidal formula was more distant from zero than that for the ellipsoidal and spherical formulas (*P* < 0.001), suggesting higher bias introduced by the cuboidal formula. APE using the extracanalicular component was significantly lower than that using the whole tumor for calculation in the cuboidal and spherical formulas (both *P* < 0.05) but not in the ellipsoidal formula (*P* = 0.32). PE using the extracanalicular component was significantly lower than that using whole tumor for calculation in all formulas (all *P* < 0.001). In addition, the ellipsoidal and spherical formulas significantly underestimated tumor size using the extracanalicular component for calculation in contrast to overestimation of tumor size using the whole tumor for calculation.

**Fig 7:**
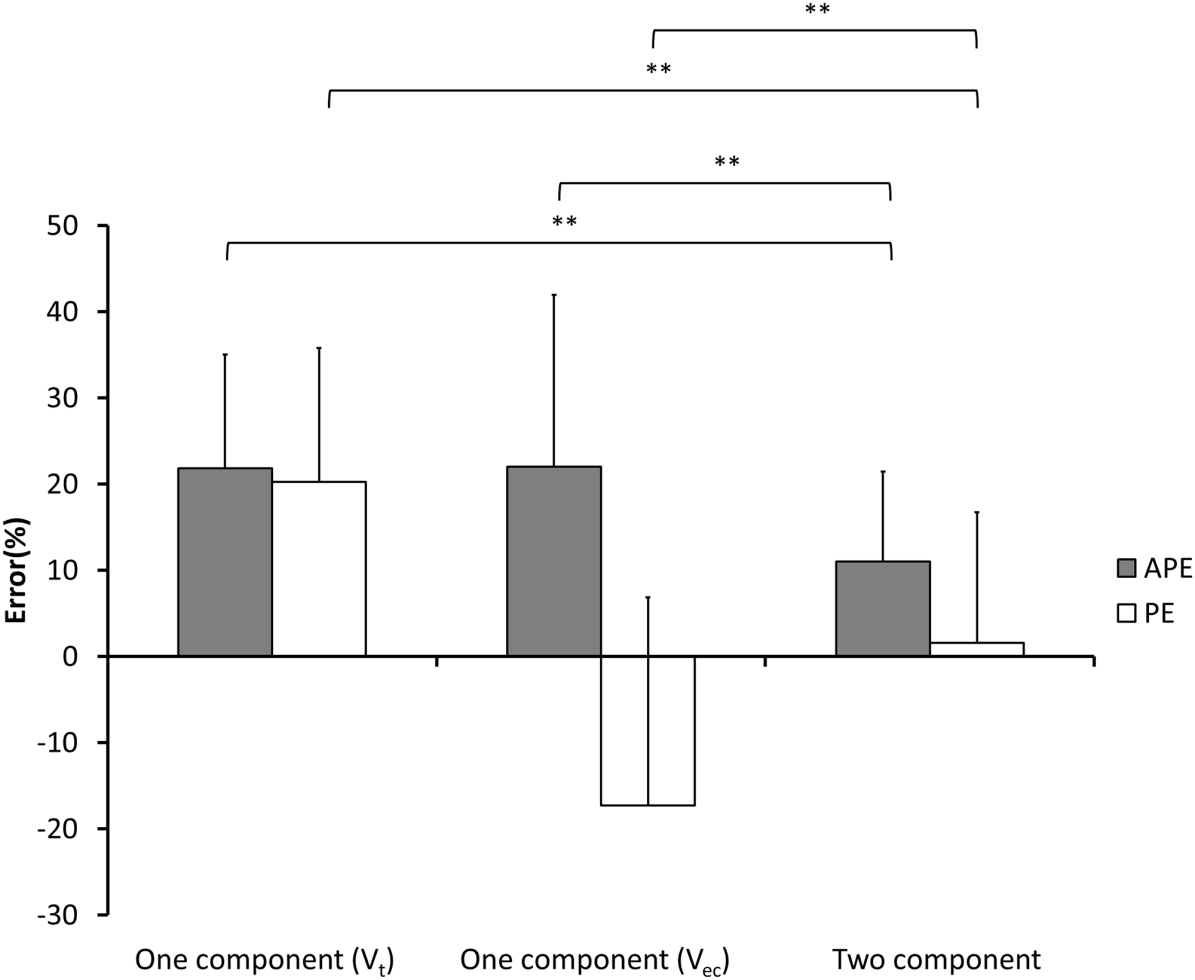
Comparisons of estimation errors among one-component ellipsoidal formulas using whole tumor and extracanalicular components of tumor, and two-component ice cream cone formula regarding (a) APE and (b) PE. Note: **P < 0.001; V_t_, volume of the whole tumor; V_ec_, volume of the extracanalicular component of tumor.

In the two-component model, the cuboidal formula showed significantly higher APE and PE than the ellipsoidal, Linskey’s, and ice cream cone formulas (all *P* < 0.001). The ice cream cone formula showed similarly lower APE and PE as the ellipsoidal and Linskey’s formulas. A comparison of APE and PE between one- and two-component formulas was shown in Fig 7. Compared to the one-component model, the two-component model using the ice cream cone formula had significantly lower APE regardless of whether the whole tumor or the extracanalicular component was used for calculation (both *P* < 0.001). Taking PE into consideration, the one-component model using the whole tumor for calculation overestimated tumor size significantly than the two-component model using the ice cream cone formula (*P* < 0.001). On the contrary, the one-component model using the extracanalicular component for calculation underestimated tumor size significantly more than did the ice cream cone formula (*P* < 0.001).

In the 17 cases of non-enhanced MR imaging, smaller size and higher APE and PE of the ice cream cone method were found (volume 3.0 ± 4.1 mL, APE 12.3 ± 12.5% and PE 6.2 ± 16.6%) than that of the other 83 cases with contrast-enhanced images (volume 5.2 ± 7.2 mL, APE 10.7 ± 10.0%, and PE 0.7 ± 14.8%) but without statistical significance (*P* = 0.229, 0.569, 0.172 for volume, APE, and PE, respectively).

### Relationship between estimation errors (APE and PE) and tumor size

In the one-component model using the extracanalicular component to represent the tumor, APE was correlated significantly positively with standard tumor volume (*y* = 3.135*x* + 50.126; *r*^2^ = 0.334, *P* < 0.001) using the cuboidal formula, and significantly negatively with standard tumor volume using the ellipsoidal (*y* = -1.317*x* + 30.978, *r*^2^ = 0.186, *P* < 0.001) and spherical (*y* = -1.070*x* + 27.162, *r*^2^ = 0.132, *P* < 0.001) formulas. There was no correlation between APE and standard tumor volume (*P* = 0.16–0.90) in all formulas based on the one-component model using the whole tumor for calculation and all formulas based on the two-component model.

PE was correlated significantly positively with standard tumor volume using the cuboidal (*y* = 3.917*x* + 38.255, *r*^2^ = 0.305, *P* < 0.001), ellipsoidal (*y* = 1.959*x* − 30.873, *r*^2^ = 0.305, *P* < 0.001) and spherical (*y* = 1.905*x* − 26.443, *r*^2^ = 0. 285, *P* < 0.001) formulas in the one-component model with the extracanalicular component used to represent the tumor, as well as using the Linskey’s (*y* = 0.646*x* + 3.994, *r*^2^ = 0. 294, *P* = 0.003) and ice cream cone (*y* = 0.815*x* − 2.321, *r*^2^ = 0. 113, *P* < 0.001) formulas in the two-component model.

## Discussion

Planimetry is rather time-consuming and manpower demanding especially for large tumors [10] although it has served as the standard method in many studies of volumetric measurement of vestibular schwannomas [10, 13, 17, 20, 21, 23, 29–32]. Our previous study has shown significantly longer measurement time for planimetry (551 ± 330 seconds) than for ellipsoid formula (48 ± 9 seconds) on thin-sliced MR images [33]. Therefore, estimation of tumor volume is considered beneficial for obtaining volume information of vestibular schwannoma faster and easier than planimetry in daily practice. Several methods have been increasingly applied to estimate the volume of vestibular schwannomas, including cuboidal [16, 17, 28], spherical [18, 24–26], and ellipsoidal [13–15, 19–23, 34] formulas no matter using either one- [16, 19, 23] or two-component models [18, 24–28]. In our study, an ice cream cone method was proposed to estimate the volume of vestibular schwannoma. Our study further compared results of the ice cream cone formula to those of the cuboidal, spherical, and ellipsoidal formulas based on one- and two-component models. Our study demonstrated that all formulas gave high and positive correlations with planimetry in estimating vestibular schwannoma volume regardless of whether the one- or two-component model was used. Our results are consistent with those of Yu et al. [13], which showed a high correlation between the ellipsoidal (ABC/2) formula and planimetry. In addition, our study also examined the intraobserver reproducibility and interobserver agreement of estimating methods, showing sufficiently high intraobserver reproducibility and interobserver agreement both qualitatively and quantitatively.

In the Bland-Altman analysis, it is not surprised that the inter- and intraobserver variations were higher in smaller size tumor due to the limitation of the minimal unit of measurement (0.01cm for diameter and 0.01mL for volume). The 95% limits of intraobserver agreements of planimetry were ±11.2%, which were within, and even better than, the range of previous studies [15, 31]. The estimating formulas, such as the ABC/2 formula and ice cream cone method which were calculated by multiple linear measurements of diameters, introduced larger intra- and interobserver variances with 95% limits of intra- and interobserver agreements ranging from ±18.9 to ±28.7%. However, the variances were even comparable to those of the planimetry in previous studies (22.1% to 26%) [15, 31].

Whether the tumor volume is estimated accurately is an important issue. However, it rarely has been noticed in contrast with the increasing use of estimating formulas for vestibular schwannomas. For example, Abaza et al. [16] used the cuboidal formula to determine the tumor volume of vestibular schwannomas using the one-component model in 1996 and Baser et al. [28] used the two-component model in 2002 without any concern for its inaccuracy in volume estimation. It was until 2012 when Walz et al. [17] cubed the maximal linear dimension and concluded it to be inaccurate for volume estimation. We found that the formula used by Walz et al. overestimates the tumor volume by a factor of 6.8 on a meta-analysis. Our results showed a significant overestimation of the tumor volume using the cuboidal formula by a factor of 1.9 to 2.4 using the one-component model and 2.1 using the two-component model. The method of Walz et al. [17] overestimates the tumor size even more than the cuboidal formula by multiplying three orthogonal diameters of the tumor as in our study. The ellipsoidal formula (also called the ABC/2 formula) has been applied increasingly to estimate the volume of vestibular schwannomas since 2000 [13–15, 17, 19–22]. The ellipsoidal formula has been shown to overestimate the volume of a non-ellipsoidal object, such as an irregularly shaped intracerebral hematoma [35–38]. Prior studies have shown a wide variation of overall estimation errors ranging from 37% overestimation [19] to 15.6% underestimation [21] for vestibular schwannoma. Such discrepancy is can be attributed partly to whether the intracanalicular component of the tumor is included or not. Theoretically, inclusion of the intracanalicular component of vestibular schwannoma will lead to overestimation of tumor volume, while its exclusion might cause underestimation of tumor volume. In our study, an overall 20.3% overestimation of tumor volume was encountered with the ellipsoidal formula when the whole tumor was estimated. On the contrary, the overall tumor volume was underestimated by 21.5% when only the extracanalicular component was used for estimation. Our results are consistent with those of prior studies showing 35% to 37% overestimation when the whole tumor was estimated [15, 19], and with those showing 4.1% to 15.6% underestimation when only the extracanalicular component was used for estimation [13, 19, 21]. Similar to the ellipsoidal formula, the spherical formula similarly overestimated the tumor volume by 28.6% when the whole tumor was estimated and underestimated the tumor volume by 17.3% when only the extracanalicular component was used for estimation.

Compared to all formulas based on the one-component model, the ice cream cone formula and other two-component formulas significantly reduced the APE. Excluding the cuboidal formula, two-component formulas reduced the APE approximately by half. The ice cream cone formula did not differ from Linskey’s formula or the ellipsoidal formula regarding either APE or PE. Our results suggest that the ice cream cone formula is equivalent to Linskey’s formula and the ellipsoidal formula and it allows for more accurate estimation of vestibular schwannoma volume than all one-component formulas regardless of whether the whole tumor or the extracanalicular component is used for estimation.

In the one-component model using the ellipsoidal and spherical formulas and using the extracanalicular component to represent the tumor, APE was correlated significantly negatively with standard tumor volume because the intracanalicular component occupied more percentage of the whole tumor when the tumor was smaller, causing more underestimation. On the other hand, there was almost always overestimation of tumor volume when the cuboidal formula was used, thus the effect of underestimation caused by using the extracanalicular component to represent the tumor could partially neutralize the overestimation of the cuboidal formula, causing lower APE. There was no correlation between APE and standard tumor volume in all formulas based on the one-component model using the whole tumor for calculation and all formulas based on the two-component model. However, PE was correlated significantly positively with standard tumor volume using the cuboidal ellipsoidal and spherical formulas in the one-component model with the extracanalicular component used to represent the tumor, as well as using the Linskey’s and ice cream cone formulas in the two-component model, which meant a tendency of overestimation in large-sized tumors and underestimation in small-sized tumors. It is assumed to be caused by two reasons. First, the tumor tends to be more irregularly-shaped when the tumor is larger, causing more overestimation. In the studies of volume estimation of intracerebral hematoma [36, 37], similar tendency were found. Second, in the two-component model, when the tumor is small, the intracanalicular component often tends to be more likely to be ellipsoidal, rather than cone-shaped, which caused underestimation of the tumor size when the cone formula was used to estimate the volume of an ellipsoid.

To our knowledge, correlation of different estimation methodologies with the exact tumor volumes of acoustic neuromas at surgery has never been reported yet. Although the planimetry was regarded as the gold standard in our study, the volume it provided was still a mathematical estimate. Although all the two-component formulas, except the cuboidal formula, showed significantly lower APE and PE than that of the one-component formulas when using planimetry as the gold standard, we did not know for sure whether this observation holds true if the exact tumor volumes at surgery were used as a reference. On the other hand, tumor volumes estimated by ellipsoidal and spherical formulas based on one-component model were not different from those estimated by two-component formulas in our study.

Some software products have been developed to allow quick volumetric measurements. However, these products are not available everywhere and often require learning to use the functions. In contrast, in the ice cream bone method, it is easy to measure four individual variables and simple to compute the tumor volume using the formula created on either Microsoft Excel, which is a popular software and easy to be used, or OpenOffice Calc, which is a free software.

Our study has four limitations. First, MRI protocols used in our study were not uniform. While some patients received thin-sliced contrast-enhanced T1WI, others received steady state imaging sequences without contrast enhancement. It is known that steady state imaging sequences demonstrate much poorer soft tissue contrast than contrast-enhanced T1WI. Nevertheless, steady state imaging sequences allow for better soft tissue-fluid contrast than contrast-enhanced T1WI for edge recognition and volume estimation. The tumor volume as well as the APEs and PEs of the ice cream cone method measured on the non-enhanced images did not differ from those measured on contrast-enhanced images in our study. Second, due to the lack of coronal thin-sliced images, the height of the lesion (dimension C) was calculated by the product of slice thickness and the number of slices containing the tumor, instead of directly measuring the height on coronal images. This may be a potential source of error. Third, the two observers of our study were residents under the supervision of the same senior neuroradiologist who judged about whether the ROI was properly drawn or not. It makes this comparison not exactly "interobserver". Nevertheless, the supervisor did not draw the ROI himself. Finally, our study was retrospective and did not provide longitudinal follow-up. A prospective study to evaluate the temporal evolution of volume of vestibular schwannoma after treatment with consideration of the accuracy and precision of volume estimating tool is warranted.

## Conclusion

The ice cream cone method and other two-component formulas including the ellipsoidal and Linskey’s formulas allow for estimation of vestibular schwannoma volume more accurately than all one-component formulas.

## Supporting information

S1 Fig Schematic of patient enrollment. (TIF)

S1 Table MRI protocol of the 100 cases of vestibular schwannomas. (DOCX)
