# Supplementary figures and images for: Vestibular schwannomas: Accuracy of tumor volume estimated by ice cream cone formula using thin-sliced MR images
Source: PLoS One. 2018 Feb 13;13(2):e0192411. doi: 10.1371/journal.pone.0192411 (PMC5810994; doi:10.1371/journal.pone.0192411)

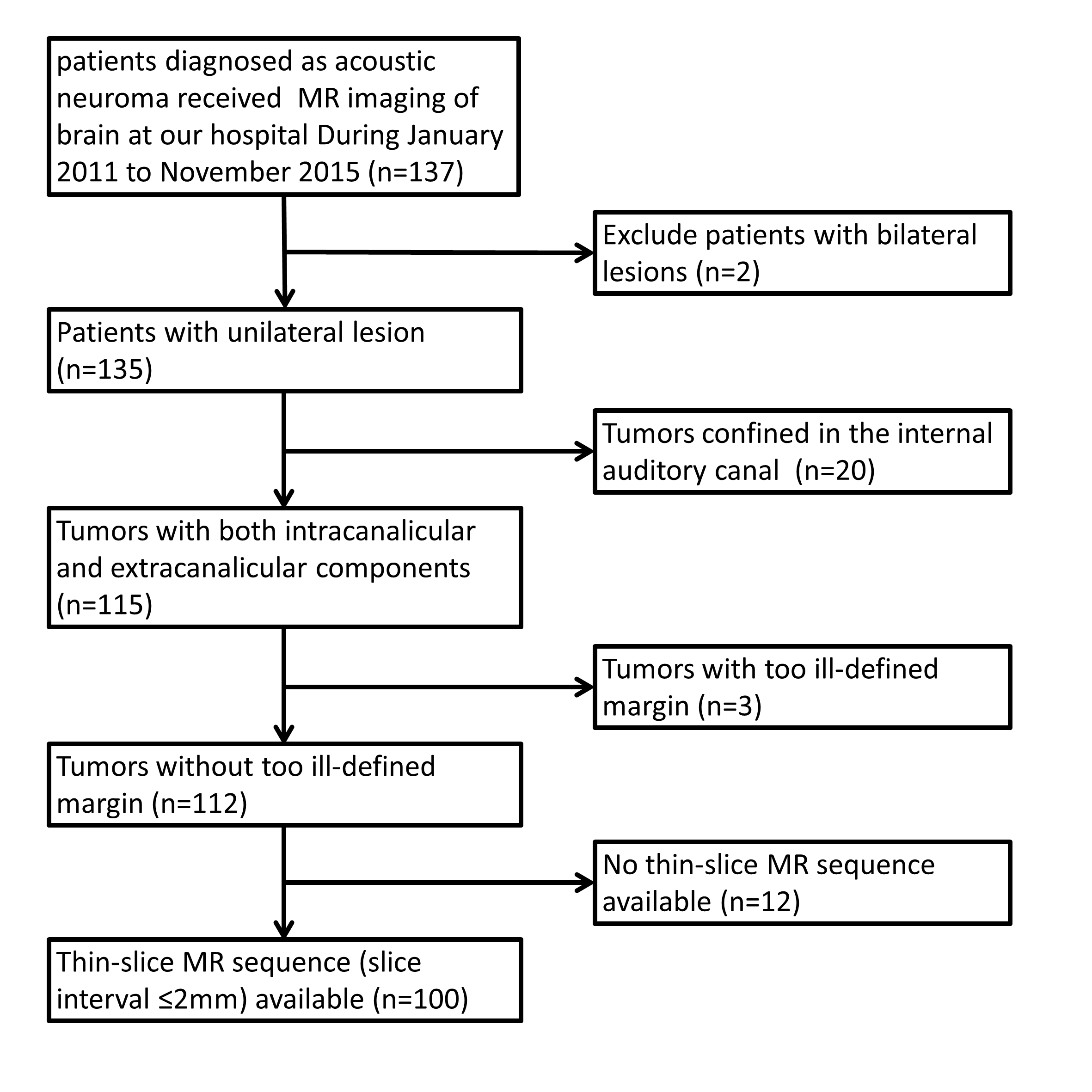

Supplement: S1 Fig — (TIF) [file pone.0192411.s001.tif]
