# Supplementary material for: Vestibular schwannomas: Accuracy of tumor volume estimated by ice cream cone formula using thin-sliced MR images
Source: PLoS One. 2018 Feb 13;13(2):e0192411. doi: 10.1371/journal.pone.0192411 (PMC5810994; doi:10.1371/journal.pone.0192411)
Supplement: S1 Table — (DOCX) [file pone.0192411.s002.docx]

**S1 Table. MRI protocol of the 100 cases of vestibular schwannomas**

| Case number | 9 | 74 | 17 |
| --- | --- | --- | --- |
| Sequence | SE T1WI | FSPGR/FFE/FLASH | FIESTA-C/bFFE |
| contrast-enhanced images | Yes | Yes | No |
| Magnetic field strength (T) | 1.5 | 1.5 or 3 | 1.5 or 3 |
| Manufacturer &  model name | GE Signa HDxt/  Philips Achieva | GE Signa HDxt/  GE Discovery MR750/  Philips Achieva/  Siemens Magnetom vision plus | GE Signa HDxt/  GE Discovery MR750/  Philips Achieva |
| Repetition time (msec) | 500~800 | 4.9~36 | 4.6~11.2 |
| Echo time (msec) | 10~12 | 1.9~5.7 | 2.2~5.6 |
| Inversion time (msec) | 0 | 0~34 | 0 |
| Flip angle (degree) | 90 | 12~40 | 50~65 |
| Field of view (mm × mm) | 320 × 320 or  280 × 280 | 220 × 220 | 220 × 220 |
| Matrix (mm × mm) | 256 × 256 | 288 × 224 or  276 × 226 | 288 × 224 or  276 × 226 |
| Voxel size (mm^3^)^*^ | 2.39 or 3.13 | 0.76~1.05 | 0.76~1.05 |
| Slice thickness (mm) | 2 | 0.9~1.4 | 0.8~1.4 |
| Dimension | 2D | 2D or 3D (ZIP by 2) | 3D (ZIP by 2) |

SE T1WI = spin echo T1-weighted imaging; FSPGR = fast spoiled gradient echo; FLASH = fast low angle shot; bFFE = balanced fast field echo; FFE = fast field echo; FIESTA-C = fast imaging employing steady-state acquisition-cycled phases; bFFE = balanced FLASH = fast field low angle shot; FSPGR = fast spoiled gradient echo; SE T1WI = spin echo T1WI; ZIP = zero interpolated.
